# Prediction Model Based on the Combination of Cytokines and Lymphocyte Subsets for Prognosis of SARS-CoV-2 Infection

**DOI:** 10.1007/s10875-020-00821-7

**Published:** 2020-07-13

**Authors:** Ying Luo, Liyan Mao, Xu Yuan, Ying Xue, Qun Lin, Guoxing Tang, Huijuan Song, Feng Wang, Ziyong Sun

**Affiliations:** 1 Department of Laboratory Medicine, Tongji Hospital, Tongji Medical College, Huazhong University of Science and Technology, Jiefang Road 1095, Wuhan, 430030 China; 2 Department of Clinical Immunology, Tongji Hospital, Tongji Medical College, Huazhong University of Sciences and Technology, Jiefang Road 1095, Wuhan, 430030 China

**Keywords:** Coronavirus disease 2019, severe acute respiratory syndrome coronavirus 2, cytokines, lymphocyte subsets, prognosis

## Abstract

**Background:**

There are currently rare satisfactory markers for predicting the death of patients with coronavirus disease 2019 (COVID-19). The aim of this study is to establish a model based on the combination of serum cytokines and lymphocyte subsets for predicting the prognosis of the disease.

**Methods:**

A total of 739 participants with COVID-19 were enrolled at Tongji Hospital from February to April 2020 and classified into fatal (*n* = 51) and survived (*n* = 688) groups according to the patient’s outcome. Cytokine profile and lymphocyte subset analysis was performed simultaneously.

**Results:**

The fatal patients exhibited a significant lower number of lymphocytes including B cells, CD4^+^ T cells, CD8^+^ T cells, and NK cells and remarkably higher concentrations of cytokines including interleukin-2 receptor, interleukin-6, interleukin-8, and tumor necrosis factor-α on admission compared with the survived subjects. A model based on the combination of interleukin-8 and the numbers of CD4^+^ T cells and NK cells showed a good performance in predicting the death of patients with COVID-19. When the threshold of 0.075 was used, the sensitivity and specificity of the prediction model were 90.20% and 90.26%, respectively. Meanwhile, interleukin-8 was found to have a potential value in predicting the length of hospital stay until death.

**Conclusions:**

Significant increase of cytokines and decrease of lymphocyte subsets are found positively correlated with in-hospital death. A model based on the combination of three markers provides an attractive approach to predict the prognosis of COVID-19.

**Electronic supplementary material:**

The online version of this article (10.1007/s10875-020-00821-7) contains supplementary material, which is available to authorized users.

## Introduction

The novel coronavirus disease 2019 (COVID-19) caused by severe acute respiratory syndrome coronavirus 2 (SARS-CoV-2) infection presents a paramount and urgent threat to global health [1]. As of 2 May 2020, there were around 3,200,000 confirmed cases, including more than 220,000 deaths, reported worldwide [2]. Therefore, combating this new virus and stopping the epidemic are a matter of urgency. Given that understanding the status of the disease is essential for providing the best possible care for patients, exploring potential markers for predicting the mortality is crucial for halting the progression of the disease [3].

In the past few months, several parameters including age, clinical symptoms, and imaging presentations have been found associated with the risk of death or severity of the illness in patients with COVID-19 [4–6]. In terms of laboratory tests, some studies described interleukin-6 (IL-6) as a biomarker associated with a high case fatality of SARS-CoV-2 infection [7–10]. Meanwhile, other cytokines including interleukin-10 and interleukin-1β also showed the correlation with disease severity [11]. Moreover, d-dimer and C-reactive protein have also been described to be predictive factors of COVID-19 disease course/mortality [12–15]. However, these previous studies are at a high risk of bias owing to a limited number of subjects and imprecise design [16]. Therefore, their performance estimates are likely to be too optimistic and not very precise.

Lymphocytes and their subsets play an important role in the maintenance of the immune system function [17]. Several studies reported that lymphocyte count had a moderate value for predicting the progress of the disease [18], and a limited number of studies explored the value of lymphocyte subsets for the prognosis of COVID-19 [11, 19, 20]. However, although these studies have suggested the potential use of lymphocyte subsets for COVID-19 prognostic purpose, other markers, either individually or in combination, have rarely been previously examined for this issue.

The current study aims to investigate the predictive value of the combination of serum cytokines and lymphocyte subsets to predict mortality in patients with SARS-CoV-2 infection.

## Methods

### Study Design

The present study was carried out at Tongji Hospital (the largest hospital in the central region of China) in Wuhan, China. Consecutive hospitalized patients with confirmed COVID-19 were recruited from February to April 2020. COVID-19 was diagnosed if patients met the following criteria: (1) having typical clinical symptoms; (2) having typical imaging findings; and (3) positive for SARS-CoV-2 real-time reverse transcription-polymerase chain reaction. The patients who died during hospitalization were defined as the fatal group. The patients who recovered during hospitalization and finally discharged were defined as the survived group. This study was approved by the ethical committee of Tongji Hospital, Tongji Medical College, Huazhong University of Science and Technology, Wuhan, China (TJ-C20200128).

### Real-time Reverse Transcription-Polymerase Chain Reaction

The clinical samples obtained from patients at admission or during the hospital stay were maintained in viral-transport medium. SARS-CoV-2 was confirmed by using TaqMan One-Step reverse transcription-polymerase chain reaction (RT-PCR) Kits from Shanghai Huirui Biotechnology Co., Ltd. and Shanghai BioGerm Medical Biotechnology Co., Ltd. Briefly, RNA was extracted from clinical samples. Five microliters of RNA was used for real-time RT-PCR, which targeted the ORF1ab and N gene. Real-time RT-PCR was performed using the following conditions: 50 °C for 15 min and 95 °C for 5 min, 45 cycles of amplification at 95 °C for 10 s and 55 °C for 45 s. The positive SARS-CoV-2 real-time RT-PCR result was defined if both ORF1ab and N cycle thresholds were < 35.

### Cytokine Profile Analysis

Serum samples were collected from study participants. The levels of interleukin-2 receptor (IL-2R), interleukin-8 (IL-8), and tumor necrosis factor-α (TNF-α) were measured according to an automatic procedure of a solid-phase two-site chemiluminescent immunometric assay via IMMULITE 1000 Analyzer (Siemens). The level of IL-6 was measured by the electrochemiluminescence method (Roche Diagnostics).

### Lymphocyte Subset Analysis

Heparinized peripheral blood was collected from study participants. The percentages and absolute numbers of CD4^+^ T cells, CD8^+^ T cells, B cells, and NK cells were determined by using TruCOUNT tubes and BD Multitest 6-color TBNK Reagent Kit (BD Biosciences) according to the manufacturer’s instructions. In brief, 50 μL of whole blood was labeled with 6-color TBNK antibody cocktail for 15 min in room temperature. After adding 450 μL of FACS Lysing Solution, the samples were analyzed with a FACSCanto flow cytometer using FACSCanto clinical software (BD Biosciences). Cells with CD45 high expression and with low side scatter were gated as lymphocytes. TruCOUNT beads were gated based on side scatter and fluorescence intensity. CD3^+^ cells in lymphocyte gate were defined as total T cells. CD4^+^CD8^−^ and CD8^+^CD4^−^ cells in CD3^+^ cells were defined as CD4^+^ T cells and CD8^+^ T cells, respectively. CD19^+^ and CD16^+^CD56^+^ cells in CD3^−^ cells were defined as B cells and NK cells, respectively.

### Statistical Analysis

Descriptive analyses of the variables were expressed as mean ± standard deviation or number (%). The comparison between continuous variables was performed using the Wilcoxon test or Mann-Whitney *U* test. The chi-square test was used for comparison of categorical data. A two-sided *α* of less than 0.05 was considered statistically significant. A prediction model for predicting the outcome of death was established by using the multivariate logistic regression method. All variables with statistical significance were taken as candidates for multivariable logistic regression analyses, and the regression equation (predictive model) was obtained. The regression coefficients of the predictive model were regarded as the weights for the respective variables, and a score for each patient was calculated. Receiver operating characteristic (ROC) analysis was performed on these scores to assess the ability for distinguishing between the fatal and survived COVID-19 patients. Area under the ROC curve (AUC), sensitivity, specificity, positive predictive value (PPV), negative predictive value (NPV), positive likelihood ratio (PLR), negative likelihood ratio (NLR), and accuracy, together with their 95% confidence intervals (CI), were calculated. Data were analyzed by using SPSS 25.0 (SPSS, Chicago, IL, USA) and GraphPad Prism version 6 (GraphPad Software, San Diego, CA, USA).

## Results

### The Clinical Characteristics of Included Patients

A total of 739 patients including 51 fatal and 688 survived patients were recruited in this study (Table 1). The demographic and clinical information is summarized in Table 1. The percentage of male subjects was significantly higher in the fatal group (66.67%) than in the survived group (48.11%). The fatal cases were significantly older than the survived patients (mean age, 68.98 years vs 59.46 years). The most common symptoms on admission were fever (54.90% for the fatal cases; 67.01% for the survived patients) and cough (56.86% for the fatal cases; 58.72% for the survived patients), followed by shortness of breath (31.37% for the fatal cases; 10.32% for the survived patients), chest tightness (15.69% for the fatal cases; 21.66% for the survived patients), and diarrhea (9.8% for the fatal cases; 18.6% for the survived patients). More fatal patients presented with shortness of breath (31.37% vs 10.32%) compared with those who survived. Comorbidities in both groups were present in nearly half of patients, with hypertension being the most common comorbidity, followed by diabetes mellitus and cardiovascular disease. The frequency of complications including diabetes mellitus (17.65% vs 7.27%), hypertension (45.10% vs 26.74%), underlying lung disease (17.65% vs 5.09%), cardiovascular disease (25.49% vs 9.30%), chronic kidney disease (9.80% vs 2.03%), and hematological malignancy (3.92% vs 0.58%) was higher in the fatal cases than in the survived patients.

**Table 1:** Demographic and clinical characteristics of study participants

Variables	Fatal (*n* = 51)	Survived (*n* = 688)	P*
Sex, male (%)	34 (66.67%)	331 (48.11%)	0.011
Age (years)			
Mean ± SD	68.98 ± 11.78	59.46 ± 15.26	< 0.001
< 50	1 (1.96%)	158 (22.97%)	< 0.001
50–59	9 (17.65%)	147 (21.37%)	0.53
60–69	16 (31.37%)	213 (30.96%)	0.951
70–79	15 (29.41%)	106 (15.41%)	0.009
> 79	10 (19.61%)	64 (9.30%)	0.018
Symptoms on admission			
Cough	29 (56.86%)	404 (58.72%)	0.795
Fever	28 (54.90%)	421 (67.01%)	0.375
Shortness of breath	16 (31.37%)	71 (10.32%)	< 0.001
Chest tightness	8 (15.69%)	149 (21.66%)	0.315
Diarrhea	5 (9.80%)	128 (18.60%)	0.114
Headache	1 (1.96%)	28 (4.07%)	0.454
Nausea and vomiting	5 (9.80%)	28 (4.07%)	0.056
Muscle ache	3 (5.88%)	78 (11.34%)	0.229
Pharyngalgia	4 (7.84%)	92 (13.37%)	0.257
Underlying condition or illness			
Diabetes mellitus	9 (17.65%)	50 (7.27%)	0.008
Hypertension	23 (45.10%)	184 (26.74%)	0.005
Underlying lung disease^†^	9 (17.65%)	35 (5.09%)	< 0.001
Cardiovascular disease	13 (25.49%)	64 (9.30%)	< 0.001
Chronic kidney disease	5 (9.80%)	14 (2.03%)	< 0.001
Chronic liver disease	3 (5.88%)	71 (10.32%)	0.308
Hematological malignancy	2 (3.92%)	4 (0.58%)	0.01
Solid tumor	3 (5.88%)	21 (3.05%)	0.271
Organ transplantation	1 (1.96%)	3 (0.44%)	0.152
Days from hospital admission to death			
Mean ± SD	18.96 ± 11.22	NA	NA
< 3	2 (3.92%)	NA	NA
3–7	5 (9.80%)	NA	NA
8–14	13 (25.49%)	NA	NA
15–30	22 (43.14%)	NA	NA
> 30	9 (17.65%)	NA	NA

Data are presented as means ± SD or numbers (percentages)

*NA*, not applicable

*Comparisons were performed between fatal and survived groups using the chi-square test or Mann-Whitney *U* test

^†^Including tuberculosis, lung tumor, and chronic obstructive pulmonary disease

### The Level of Serum Cytokines in the Fatal and Survived Patients

**Fig. 1:**
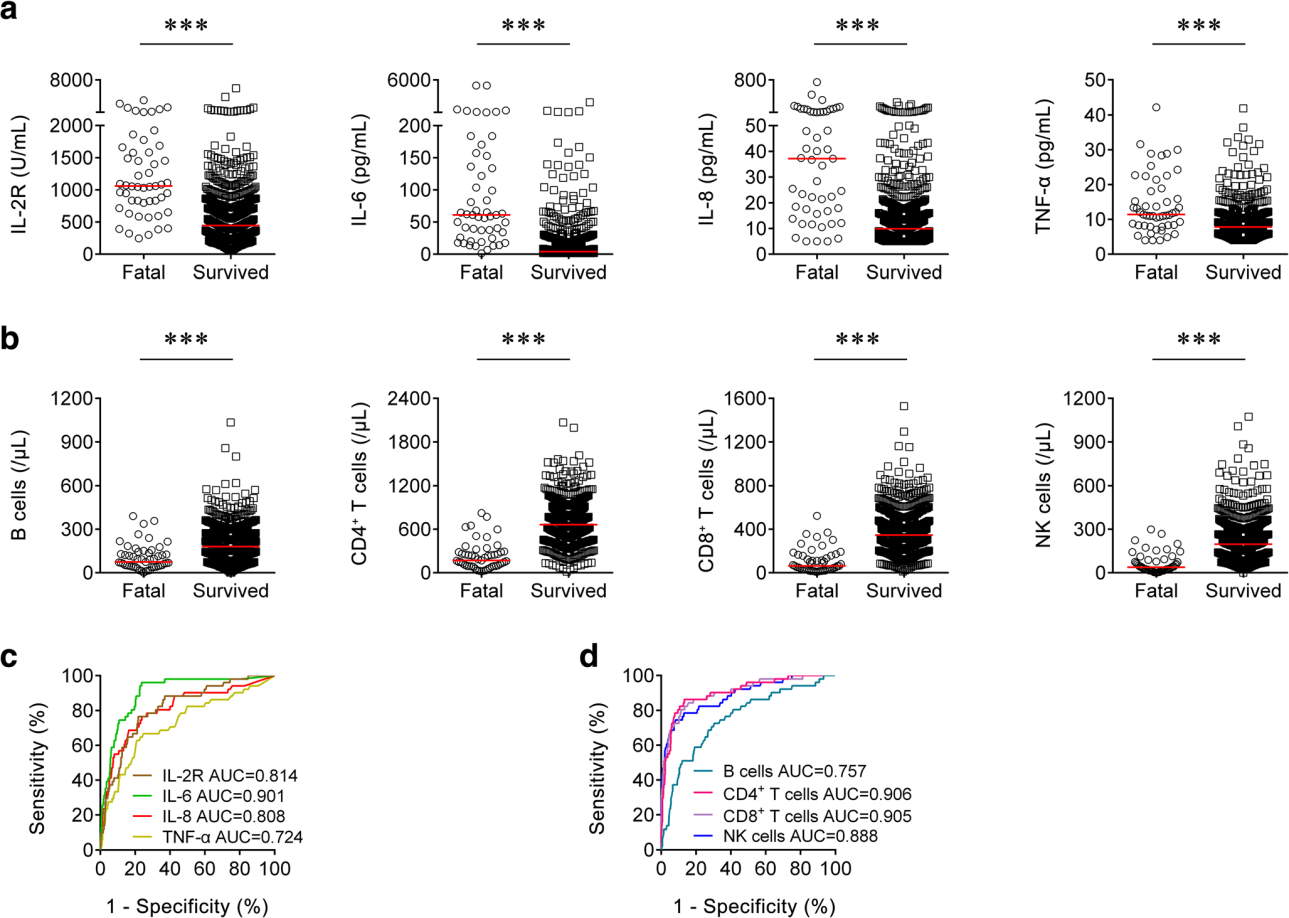
Using cytokines and lymphocyte subsets on admission for discriminating the fatal cases from the survived patients. **a** Scatter plots showing the levels of IL-2R, IL-6, IL-8, and TNF-α in the fatal cases (*n* = 51) and the survived patients (*n* = 688). Horizontal lines indicate the median. ***P < 0.001 (Mann-Whitney *U* test). **b** Scatter plots showing the numbers of B cells, CD4^+^ T cells, CD8^+^ T cells, and NK cells in the fatal cases (*n* = 51) and the survived patients (*n* = 688). Horizontal lines indicate the median. ***P < 0.001 (Mann-Whitney *U* test). **c** ROC analysis showing the performance of IL-2R, IL-6, IL-8, and TNF-α in distinguishing the fatal cases from the survived patients. **d** ROC analysis showing the performance of B cells, CD4^+^ T cells, CD8^+^ T cells, and NK cells in distinguishing the fatal cases from the survived patients. IL-2, interleukin-2 receptor; IL-6, interleukin-6; IL-8, interleukin-8; TNF-α, tumor necrosis factor-α; ROC, receiver operating characteristic curve; AUC, area under the curve

The concentrations of serum cytokines including IL-2R, IL-6, IL-8, and TNF-α were measured in both fatal and survived patients on admission. The levels of IL-2R, IL-6, IL-8, and TNF-α in the fatal cases were significantly higher than in the survived patients (Fig. 1a). If using these markers to distinguish these two conditions, the best AUC was obtained for IL-6 (0.901 (95% CI, 0.860 to 0.942)) (Table 2 and Fig. 1c). Notably, IL-6 ≥ 39.5 pg/mL produced a sensitivity of 68.63% and a specificity of 90.41%, respectively (Table 2). In addition, ROC analysis showed that the AUC of IL-2R was 0.814 (95% CI, 0.755 to 0.874), with a sensitivity of 41.18% and a specificity of 92.15% when a cutoff value of 1220 U/mL was used to differentiate the fatal cases from the survived patients (Table 2). Moreover, with a threshold of 30 pg/mL, IL-8 had an AUC of 0.808 (95% CI, 0.738 to 0.879) with a sensitivity of 54.90% and a specificity of 90.26% (Table 2).

**Table 2:** The performance of various methods for distinguishing between the fatal cases and the survived patients

Variables	Cutoff value	AUC (95% CI)	Sensitivity (95% CI)	Specificity (95% CI)	PPV (95% CI)	NPV (95% CI)	PLR (95% CI)	NLR (95% CI)	Accuracy (%)
IL-2R (U/mL)	1220	0.814 (0.755–0.874)	41.18% (27.67–54.68%)	92.15% (90.14–94.16%)	28.00% (17.84–38.16%)	95.48% (93.90–97.06%)	5.25 (3.46–7.95)	0.64 (0.51–0.80)	88.63
IL-6 (pg/mL)	39.5	0.901 (0.860–0.942)	68.63% (55.89–81.36%)	90.41% (88.21–92.61%)	34.65% (25.37–43.93%)	97.49% (96.28–98.71%)	7.15 (5.33–9.61)	0.35 (0.23–0.52)	88.90
IL-8 (pg/mL)	30	0.808 (0.738–0.879)	54.90% (41.25–68.56%)	90.26% (88.05–92.48%)	29.47% (20.31–38.64%)	96.43% (95.00–97.86%)	5.64 (4.02–7.90)	0.50 (0.37–0.68)	87.82
TNF-α (pg/mL)	14.4	0.724 (0.644–0.804)	33.33% (20.40–46.27%)	90.12% (87.89–92.35%)	20.00% (11.50–28.50%)	94.80% (93.10–96.50%)	3.37 (2.15–5.28)	0.74 (0.61–0.90)	86.20
B cells (/μL)	63	0.757 (0.685–0.828)	39.22% (25.82–52.62%)	90.70% (88.53–92.87%)	23.81% (14.70–32.92%)	95.27% (93.64–96.89%)	4.22 (2.79–6.38)	0.67 (0.54–0.84)	87.14
CD4^+^ T cells (/μL)	323	0.906 (0.860–0.952)	78.43% (67.14–89.72%)	90.41% (88.21–92.61%)	37.74% (28.51–46.96%)	98.26% (97.24–99.28%)	8.18 (6.24–10.72)	0.24 (0.14–0.40)	89.58
CD8^+^ T cells (/μL)	148	0.905 (0.857–0.953)	72.55% (60.30–84.80%)	90.99% (88.85–93.13%)	37.37% (27.84–46.90%)	97.81% (96.68–98.95%)	8.05 (6.02–10.77)	0.30 (0.19–0.47)	89.72
NK cells (/μL)	81	0.888 (0.834–0.941)	66.67% (53.73–79.60%)	90.55% (88.37–92.74%)	34.34% (24.99–43.70%)	97.34% (96.10–98.59%)	7.06 (5.22–9.54)	0.37 (0.25–0.54)	88.90
Prediction model	0.075	0.956 (0.928–0.984)	90.20% (82.03–98.36%)	90.26% (88.05–92.48%)	40.71% (31.65–49.77%)	99.20% (98.50–99.90%)	9.26 (7.25–11.83)	0.11 (0.05–0.25)	90.26

*AUC*, area under the curve; *PPV*, positive predictive value; *NPV*, negative predictive value; *PLR*, positive likelihood ratio; *NLR*, negative likelihood ratio; *CI*, confidence interval; *IL-2R*, interleukin-2 receptor; *IL-6*, interleukin-6; *IL-8*, interleukin-8; *TNF-α*, tumor necrosis factor-α

### The Number of Lymphocyte Subsets in the Fatal and Survived Patients

The numbers of lymphocyte subsets including B cells, CD4^+^ T cells, CD8^+^ T cells, and NK cells were counted in both fatal cases and survived patients on admission. Our results showed that the numbers of B cells, CD4^+^ T cells, CD8^+^ T cells, and NK cells in the fatal cases were significantly lower than in the survived patients (Fig. 1b). The AUCs of both CD4^+^ T cells and CD8^+^ T cells were over 0.9 for distinguishing between the fatal cases and the survived patients (Fig. 1d). Using the cutoff value of 323 cells/μL, the sensitivity and specificity of CD4^+^ T cells for discriminating the fatal cases from the survived patients were 78.43% and 90.41%, respectively (Table 2). With a threshold of 148 cells/μL, CD8^+^ T cell was able to distinguish the fatal cases from the survived patients with a sensitivity of 72.55% and a specificity of 90.99% (Table 2).

### Change of Cytokines and Lymphocyte Subsets in Patients Between Admission and Death or Discharge

**Fig. 2:**
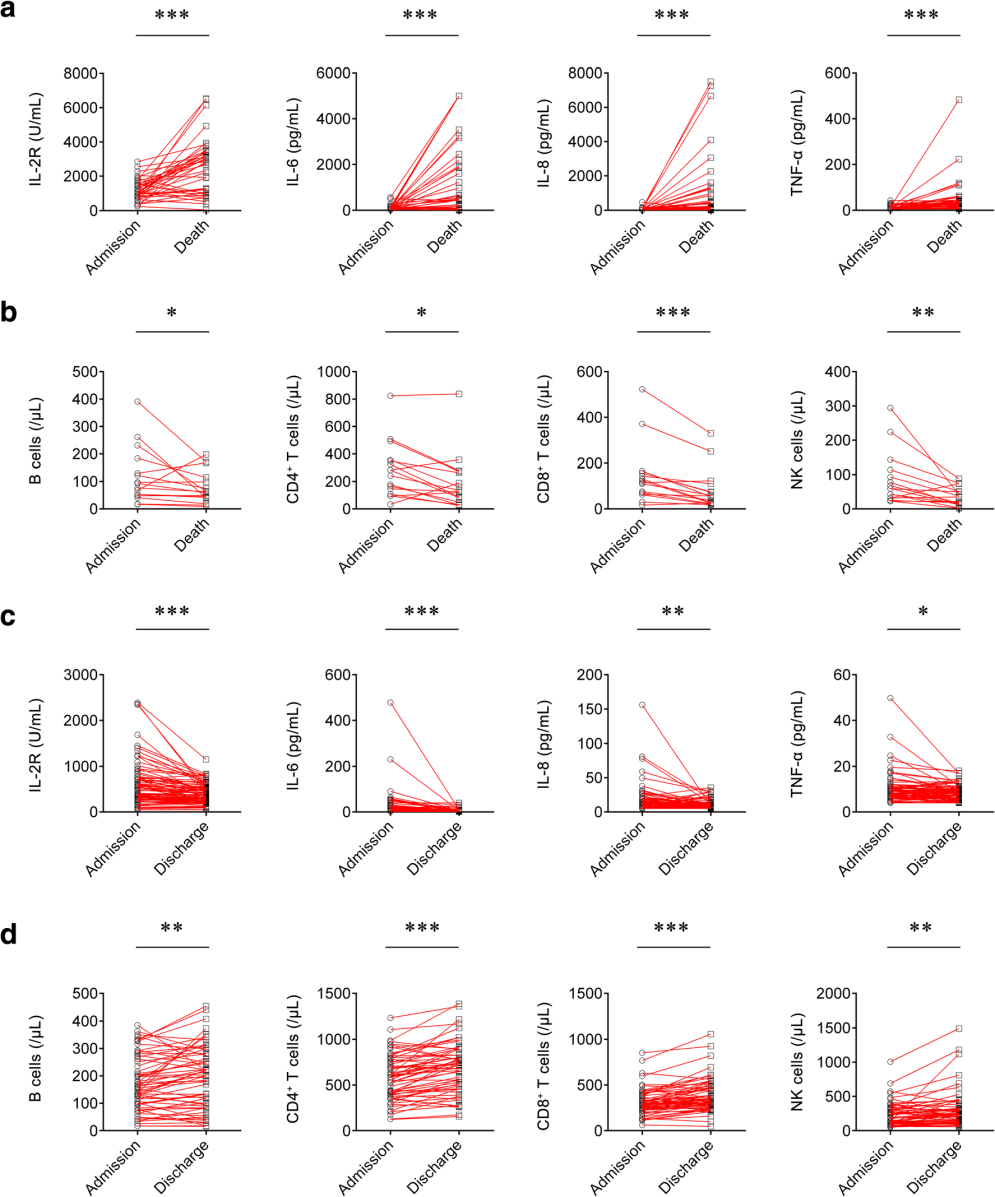
Change of cytokines and lymphocyte subsets in the same patients. **a** Line graphs showing the levels of IL-2R, IL-6, IL-8, and TNF-α for each fatal patient on admission and death (*n* = 42). One line represents one patient. ***P < 0.001 (Wilcoxon’s test). **b** Line graphs showing the numbers of B cells, CD4^+^ T cells, CD8^+^ T cells, and NK cells for each fatal patient on admission and death (*n* = 16). One line represents one patient. *P < 0.05, **P < 0.01, ***P < 0.001 (Wilcoxon’s test). **c** Line graphs showing the levels of IL-2R, IL-6, IL-8, and TNF-α for each survived patient on admission and discharge (*n* = 86). One line represents one patient. *P < 0.05, **P < 0.01, ***P < 0.001 (Wilcoxon’s test). **d** Line graphs showing the numbers of B cells, CD4^+^ T cells, CD8^+^ T cells, and NK cells for each survived patient on admission and discharge (*n* = 62). One line represents one patient. **P < 0.01, ***P < 0.001 (Wilcoxon’s test). IL-2, interleukin-2 receptor; IL-6, interleukin-6; IL-8, interleukin-8; TNF-α, tumor necrosis factor-α

We compared cytokines and lymphocyte subsets in the fatal cases between admission and death. The levels of IL-2R, IL-6, IL-8, and TNF-α were significantly increased at the time of death compared with those at the time of admission (Fig. 2a). On the contrary, the numbers of B cells, CD4^+^ T cells, CD8^+^ T cells, and NK cells were significantly decreased at the time of death compared with those at the time of admission (Fig. 2b). Furthermore, the concentrations of cytokines and the numbers of lymphocyte subsets were compared between admission and discharge in the survived patients. Differently, the survived patients showed lower levels of IL-2R, IL-6, IL-8, and TNF-α and higher numbers of B cells, CD4^+^ T cells, CD8^+^ T cells, and NK cells on discharge compared with those on admission (Fig. 2c, d).

### Correlation Between Cytokines and Lymphocyte Subsets on Admission and Length of Hospital Stay Until Death

**Fig. 3:**
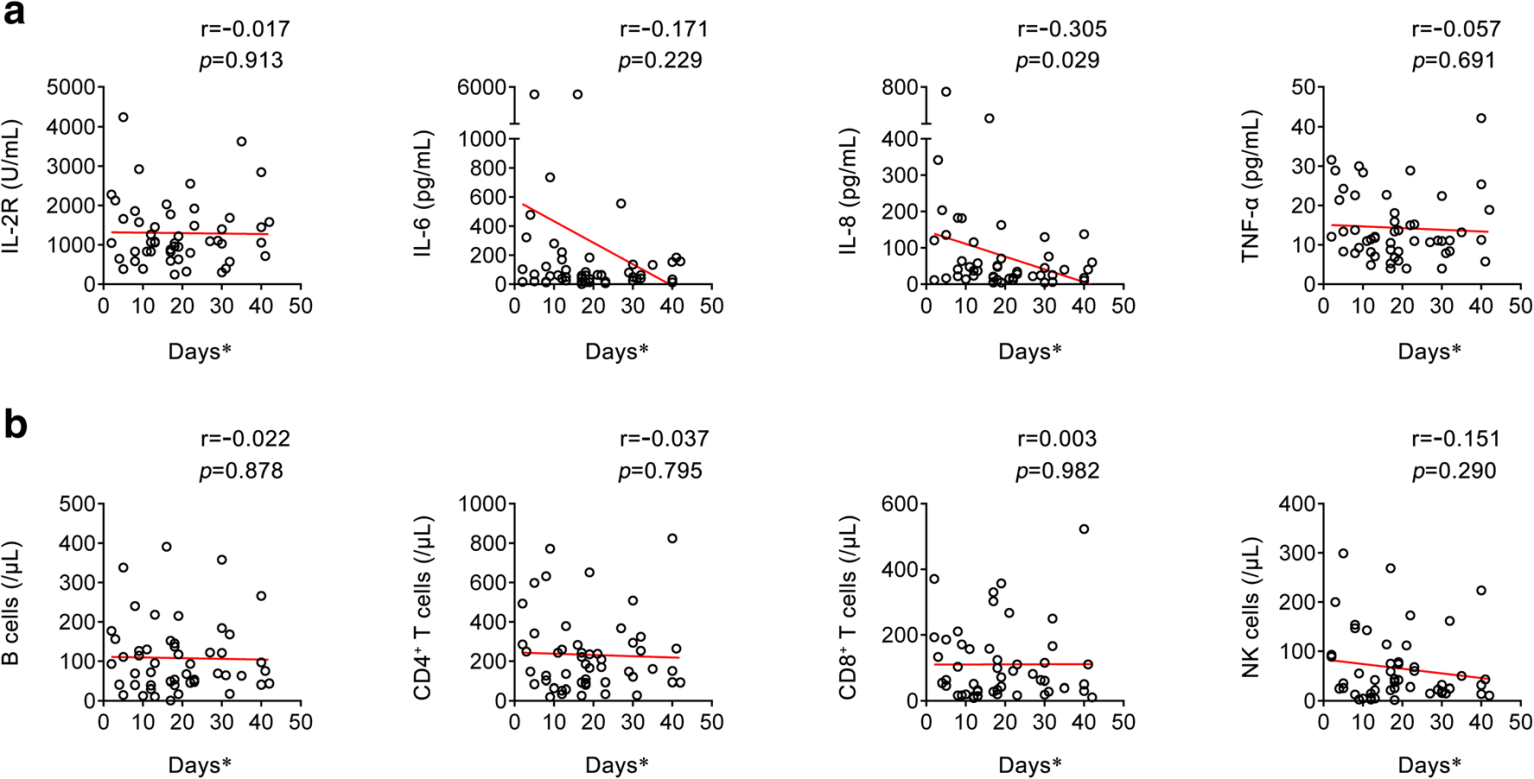
Correlation between cytokines and lymphocyte subsets on admission and days from hospital admission to death in the fatal patients. **a** Correlation between the levels of IL-2R, IL-6, IL-8, and TNF-α and days from admission onset to death (*n* = 51). **b** Correlation between the numbers of B cells, CD4^+^ T cells, CD8^+^ T cells, and NK cells and days from admission onset to death (*n* = 51). Each symbol represents an individual fatal patient. *Days from admission onset to death. IL-2, interleukin-2 receptor; IL-6, interleukin-6; IL-8, interleukin-8; TNF-α, tumor necrosis factor-α

We further analyzed the correlation between the concentration of cytokines on admission and length of hospital stay until death in the fatal group. Interestingly, there was a significant negative correlation between the level of IL-8 and length of hospital stay until death, but no significant correlation was found between the levels of IL-2R, IL-6, and TNF-α and days from admission onset to death (Fig. 3a). Moreover, no significant correlation was found between the numbers of B cells, CD4^+^ T cells, CD8^+^ T cells, and NK cells on admission and length of hospital stay until death (Fig. 3b).

### Establishing the Model for Predicting the Death of Patients with COVID-19

**Fig. 4:**
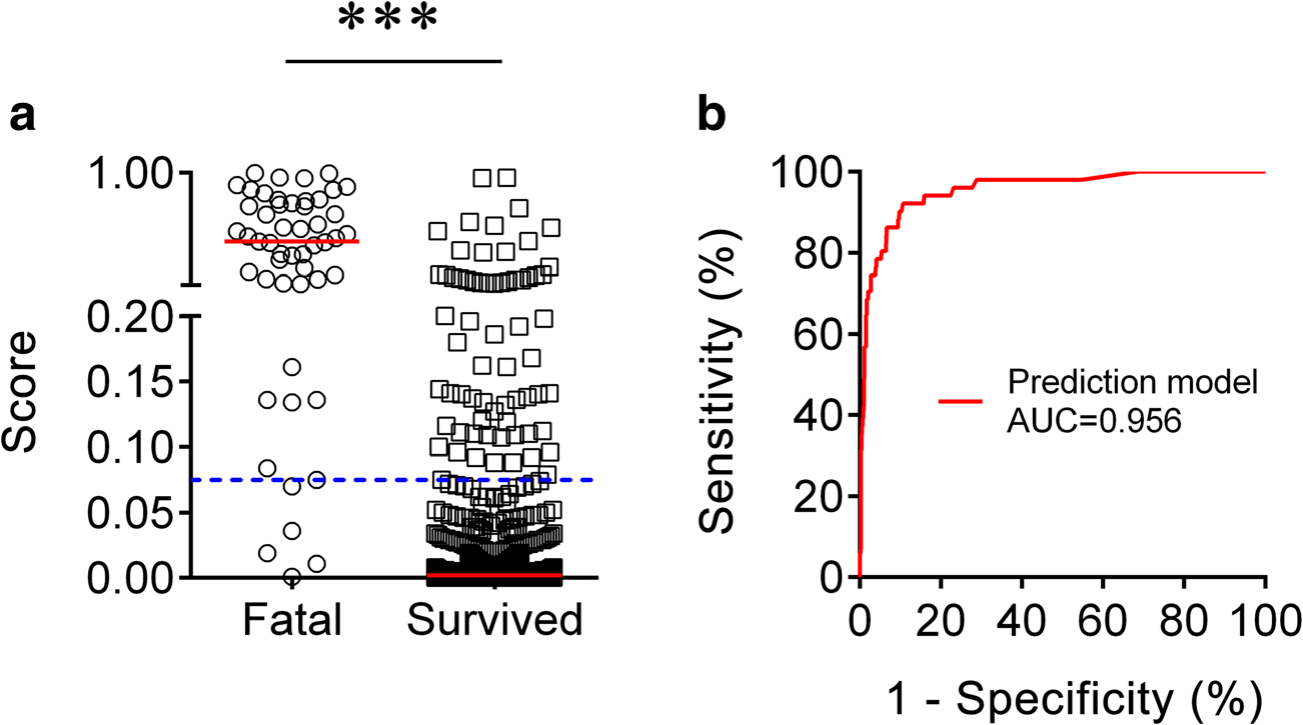
Establishment of prediction model for prognosis of SARS-CoV-2 infection based on combination of cytokines and lymphocyte subsets. **a** Scatter plots showing the score of prediction model in the fatal cases (*n* = 51) and the survived patients (*n* = 688). Horizontal lines indicate the median. ***P < 0.001 (the Mann-Whitney *U* test). Blue dotted lines indicate the cutoff value in distinguishing these two groups. **b** ROC analysis showing the performance of prediction model in distinguishing the fatal cases from the survived patients. ROC, receiver operating characteristic curve; AUC, area under the curve

To establish a prediction model based on the combination of cytokines and lymphocyte subsets on admission for distinguishing the fatal cases from the survived patients, all variables with statistical significance were used for multivariable logistic regression analysis. A prediction model was built as follows: *P* = 1/[1 + e^−(0.017 × IL-8 (pg/mL) − 0.006 × CD4 T cells (cells/μL) − 0.016 × NK cells (cells/μL) + 1.084)^], where *P* is the predictive value and *e* is the natural logarithm. ROC analysis showed that the AUC of the prediction model was 0.956 (95% CI, 0.928 to 0.984) (Fig. 4). When the cutoff value was set at 0.075, the following diagnostic parameters of the model were obtained: sensitivity, 90.20% (95% CI, 82.03 to 98.36%); specificity, 90.26% (95% CI, 88.05 to 92.48%); and accuracy, 90.26% (Table 2). These data suggested that our established model based on the combination of three markers had a good performance for predicting the death of patients with COVID-19.

## Discussion

COVID-19 has spread worldwide as an emerging infectious disease. Risk factors for the possibility of mortality of this disease have not yet been well and fully delineated [21]. Although substantial efforts have been made to develop novel methods for COVID-19 prognosis, emerging approaches still need further corroboration [22–24]. As a result, there are scarce effective tools to meet the clinical requirements for predicting the prognosis of COVID-19.

A model for the early prediction of the death of patients with COVID-19 has not been thoroughly investigated yet. We present a large-scale study comparing serum cytokines and lymphocyte subsets between the fatal cases and the survived patients. The present study identified several risk factors for death in patients who were hospitalized with COVID-19. Notably, we observed significant increases of serum cytokines and decreases of lymphocyte subsets in the fatal cases compared with the survived patients. Based on the combination of three markers, we successfully built an optimal prediction model with good utility. Our established model provides a promising and viable tool in predicting mortality in patients with SARS-CoV-2 infection. Furthermore, we found that the concentration of IL-8 on admission has the potential to predict the length of hospital stay until death.

In addition, opposite trends were found in cytokines and lymphocyte subsets in the fatal and survived groups. The levels of cytokines gradually increased, but the numbers of lymphocyte subsets gradually decreased with growing hospital stay in the fatal cases (Supplementary Fig. 1A, B). By contrast, for the survived patients, the concentrations of cytokines gradually decreased, and the numbers of lymphocyte subsets gradually returned to normal level as body’s immunity gradually recovered (Supplementary Fig. 1C, D). These data suggest that the dynamic monitoring of cytokines and lymphocyte subsets provides a potential value for mastering the process of the disease, but this remains to be elucidated in more detail.

Recent studies indicated that cytokine storm was implicated in SARS-CoV-2 infection and served as a cause for deleterious consequence [25, 26]. In line with prior studies, we found that patients with COVID-19, especially those with poor prognosis, were in a state of hyper-inflammation and poor immune function in the early stage of the disease. We speculate that there is an inextricable intrinsic connection between the immunity and cytokine secretion represented by lymphocytes in patients with SARS-CoV-2 infection. If the patient cannot receive appropriate treatment and reverse the condition in time, the patient would be at a high risk of poor prognosis. Our findings provide potential help in improving the treatment and management of COVID-19. However, more evidences are needed to address this issue.

Two limitations should be mentioned. First, cytokine and lymphocyte subset analyses are not routine laboratory tests, which would limit the usage of the prediction model. Second, this is a single-center study with a limited sample size, especially for the fatal group. Thus, a multi-center design with larger cohort is warranted in the future.

In summary, our study demonstrates that high levels of cytokines and low numbers of lymphocyte subsets are associated with a greater risk of death for COVID-19. The prediction model established upon IL-8 and the number of CD4^+^ T cells and NK cells has a good value to distinguish the fatal cases from the survived patients among patients with COVID-19. Application of this model might be beneficial to delay the progression of the disease.

## Electronic Supplementary Material

Supplementary Figure 1 Dynamic monitoring of cytokines and lymphocyte subsets in the representative patient from the fatal and survived groups. (A) Line diagrams showing the levels of IL-2R, IL-6, IL-8, and TNF-α in one representative patient from the fatal group. (B) Line diagrams showing the numbers of B cells, CD4+ T cells, CD8+ T cells, and NK cells in one representative patient from the fatal group. (C) Line diagrams showing the levels of IL-2R, IL-6, IL-8, and TNF-α in one representative patient from the survived group. (D) Line diagrams showing the numbers of B cells, CD4+ T cells, CD8+ T cells, and NK cells in a representative patient of the survived group. IL-2, interleukin-2 receptor; IL-6, interleukin-6; IL-8, interleukin-8; TNF-α, tumor necrosis factor-α. (PNG 18564 kb)

High Resolution Image (TIF 10625 kb)
