# Supplementary figures and images for: Prediction Model Based on the Combination of Cytokines and Lymphocyte Subsets for Prognosis of SARS-CoV-2 Infection
Source: J Clin Immunol. 2020 Jul 13;40(7):960–9. doi: 10.1007/s10875-020-00821-7 (PMC7357264; doi:10.1007/s10875-020-00821-7)

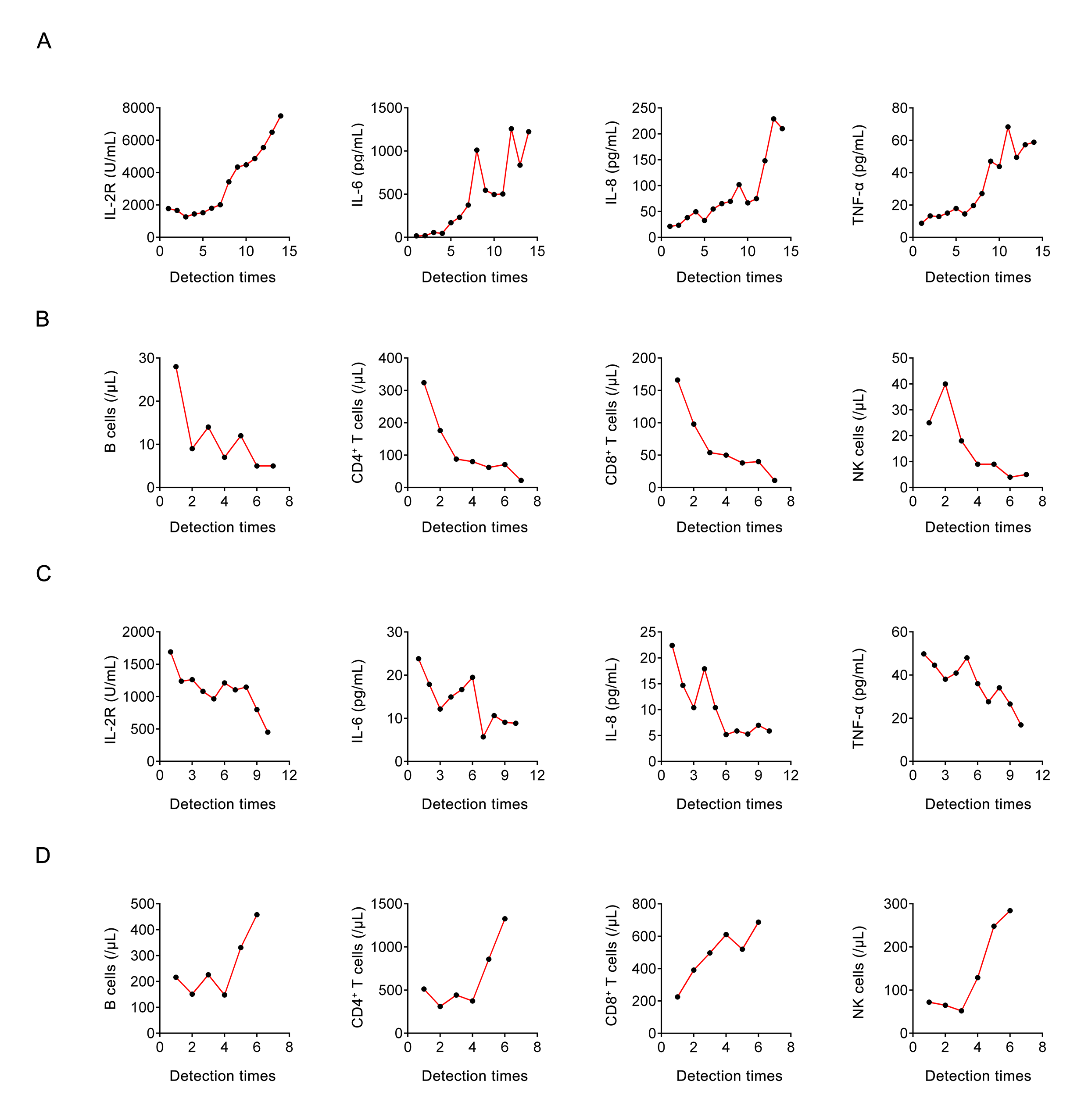

Supplement: Supplementary file 1 — Dynamic monitoring of cytokines and lymphocyte subsets in the representative patient from the fatal and survived groups. (A) Line diagrams showing the levels of IL-2R, IL-6, IL-8, and TNF-α in one representative patient from the fatal group. (B) Line diagrams showing the numbers of B cells, CD4+ T cells, CD8+ T cells, and NK cells in one representative patient from the fatal group. (C) Line diagrams showing the levels of IL-2R, IL-6, IL-8, and TNF-α in one representative patient from the survived group. (D) Line diagrams showing the numbers of B cells, CD4+ T cells, CD8+ T cells, and NK cells in a representative patient of the survived group. IL-2, interleukin-2 receptor; IL-6, interleukin-6; IL-8, interleukin-8; TNF-α, tumor necrosis factor-α. (PNG 18564 kb) [file 10875_2020_821_Fig5_ESM.png]
